# Electrochemical Degradation of Molecularly Imprinted Polymers for Future Applications of Inflammation Sensing in Cochlear Implants

**DOI:** 10.1021/acsomega.4c02906

**Published:** 2024-05-24

**Authors:** Minh-Hai Nguyen, Adrian Onken, Jan Sündermann, Madina Shamsuyeva, Pankaj Singla, Tom Depuydt, Marloes Peeters, Patrick Wagner, Konrad Bethmann, Julia Körner, Hans-Josef Endres, Thomas Lenarz, Theodor Doll

**Affiliations:** † Department of Otolaryngology and Cluster of Excellence “Hearing4all”, Hannover Medical School, Carl-Neuberg-Straße 1, 30625 Hannover, Germany; ‡ Department of Chemical Safety and Toxicology, Fraunhofer Institute of Toxicology and Experimental Medicine ITEM, Nikolai-Fuchs-Straße 1, 30625 Hannover, Germany; § IKK - Institute of Plastics and Circular Economy, Leibniz University Hannover, An der Universität 2, 30823 Garbsen, Germany; ∥ Engineering Department, University of Manchester, Engineering A building, Booth E Street, M13 9QS Manchester, United Kingdom; ⊥ Laboratory for Soft Matter and Biophysics, KU Leuven, Celestijnenlaan 200D, Leuven B-3001, Belgium; # Department of Information processing, Leibniz University Hannover, Welfengarten 1, 30167 Hannover, Germany; ∇ Institute of Electrical Engineering and Measurement Technology, Leibniz University Hannover, Appelstraße 9a, 30167 Hannover, Germany

## Abstract

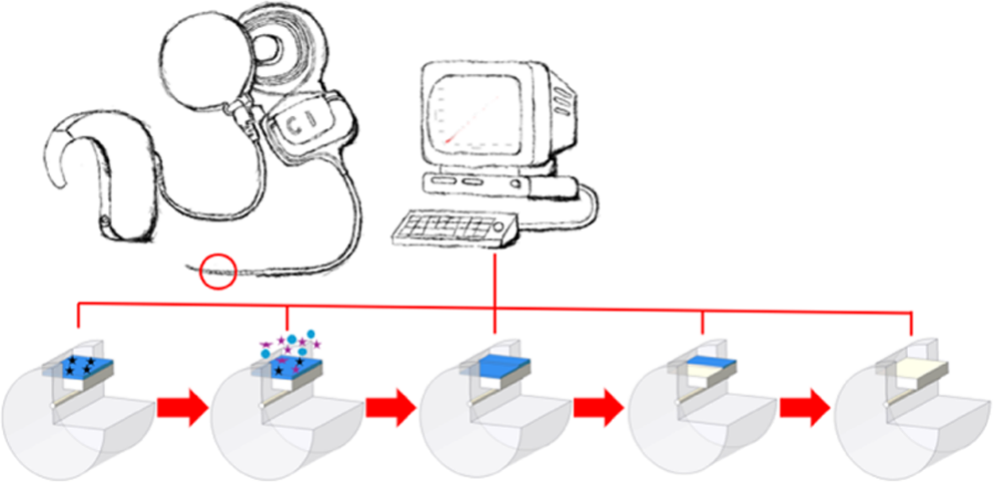

After cochlear implant (CI) insertion, there is a possibility of postoperative inflammation, which may involve proinflammatory markers such as interleukin-6. Detecting this inflammation promptly is crucial for administering anti-inflammatory drugs, if required. One potential method for detecting inflammation is using molecular imprinted polymers (MIPs). These MIPs, which can be deposited on the CI electrode, provide readout employing impedance measurements, a feature already available on the CI circuit. MIPs designed for this purpose should possess biocompatibility, conductivity, and degradability. The degradability is crucial because there is a limitation on the number of electrodes available, and once the inflammation sensor degrades after the acute inflammation period, it should remain usable as a regular electrode. In this work, conductive poly(3,4-ethylenedioxythiophene) polystyrenesulfonate-based MIPs were synthesized against biotin as a surrogate target marker. Specific biotin binding with MIPs was determined before and after degradation using electrochemical impedance spectroscopy (EIS) and compared with the control nonimprinted polymers (NIPs). Subsequently, MIPs were electrochemically degraded by EIS with different potentials, wherein a potential dependence was observed. With decreasing potential, fewer dissolved polymers and more monomer molecules were detected in the solution in which degradation took place. At a potential of 0.205 V a negligible amount of dissolved polymer in addition to the dissolved monomer molecules was measured, which can be defined as the limiting potential. Below this potential, only dissolved monomer molecules are obtained, which enables renal clearance. Biocompatibility testing revealed that both the polymer and the solution with dissolved monomer molecules do not exceed the ISO 10993–5 cytotoxicity threshold. Based on these findings, we have developed conductive, biocompatible, and controllably degradable MIPs capable of detecting biotin. This research work paves the way for the advancement of CIs, where inflammation can be detected using molecular imprinting technology without compromising the stability and biosafety of the product.

## 1 Introduction

Cochlear implants (CIs) are electronic prostheses and allow restoration of hearing in case of severe sensorineural hearing loss. In most cases of deafness, the degeneration of hair cells in the cochlear cortex leads to the deterioration of auditory input transmission to the auditory nerve. CIs replace this functionality by electrically stimulating the cochlear region via an electrode array. Platinum and silicone rubber are the most commonly used materials for implants and the electrode shaft, which is slid into the scala tympani through an opening of the inner ear’s round window. Statistically, 40% of CI devices fail between 2 and 30 years after implantation. Among all phases of CI treatment, the postsurgical healing phase is the most critical, as inflammation reactions inside the cochlea may occur between the first day after surgical intervention and up to 6 weeks thereafter. Inflammation, including that caused by direct insertion trauma, leads to scarring (fibrosis) and thus to suboptimal CI performance.^1−5^ In most cases, the CI must be removed, requiring further surgery leading to further risk, costs, and waiting time for the patient. Therefore, information about inflammation is crucial for the timely administration of anti-inflammatory medication or to facilitate surgical planning. There are currently different research approaches that target the detection of inflammation e.g., perilymph analysis during CI surgery.^6,7^ Since inflammation can occur up to 6 weeks after surgery, the accuracy of such analysis is limited. Therefore, molecularly imprinted polymers (MIPs) have been proposed as sensors for timely inflammation marker detection in body fluids on CI, as it is possible to deposit MIPs on the CI electrode.^8−11^ Since MIP sensors for implants would remain in the body for years, high standards must be fulfilled with regard to biocompatibility. In addition, the polymer layer must be conducive to improve sensitivity or enable the highest possible detection for a small amount of target molecules. MIP deposition is usually accomplished via electrochemical polymerization with electrically conductive polymers such as poly(3,4-ethylenedioxythiophene) polystyrenesulfonate (PEDOT:PSS) and polypyrrole, which have been widely researched in the literature and are reported to have excellent biocompatibility.^9,12−26^ It is also necessary that the polymer should degrade because the CI should stimulate normally again after the inflammation detection phase. Conductive polymers have so far only been degraded by overoxidation.^9,27−35^ Most overoxidation occurs in a liquid environment, with the amount of nucleophile (solvent or counterions e.g., water, hydroxide, methanol, or halides) playing an important role.^28,30^ PEDOT can overoxidize at a voltage between +0.8 and +1.1 V.^27^ However, the degraded polymeric chain is not short enough to be considered a monomer/oligomer molecule or dissolved in body fluids. Monomer molecules have a low molecular weight compared to polymer molecules, which allows the monomer molecules to be excreted in the body via the renal system.^36^ Although the conductive polymers PEDOT or polypyrrole are biocompatible according to the literature, the insoluble polymer in the body fluid can cause a foreign body reaction. Studies of the anodic degradation or overoxidation of PEDOT:PSS are very limited. In contrast, the oxidative degradation pathway of polythiophene, which like PEDOT has a thiophene group, was studied in detail.^29^ Other approaches for the degradation of conductive polymers can also be found in the literature. In most cases, the degraded polymer fragments are not short enough. In addition, the production of conductive polymers with short chains or electrode surface modification for MIP application are much more complicated compared to electrochemical polymerization.^28,36−46^

Although MIPs have been very well researched and have excellent conductivity and biocompatibility, there are currently no *in vivo* MIPs available for implants. The reasons for this are either complicated production as mentioned above, the reliable reproducibility, or the degraded polymer chain, which is not excreted by the body. Since a voltage dependence on the amount of degraded polymer chain was observed during overoxidation, our hypothesis is that monomer molecules are obtained at voltages lower than 0.8 V, which enables renal clearance. As the conductive polymer also exhibits excellent biocompatibility, it is reasonable to conclude that the degraded monomer molecules are also biocompatible. However, there is currently no literature on biocompatibility testing of the degraded monomer molecules.

In this study, we first demonstrated that the deposited layer was MIPs specific for biotin. Biotin served as a surrogate template for interleukin-6 because biotin exhibits similarity to the amino acid sequence of interleukin-6 and its epitope. The synthesized MIPs were deposited by electrodeposition of the conductive monomer 3,4-ethylenedioxythiophene (EDOT) and the counteranion poly(sodium 4-styrenesulfonate) on a platinum electrode. Afterward, MIPs and nonimprinted polymers (NIPs) based on the conductive polymer PEDOT:PSS were systematically degraded in a controlled manner using electrochemical impedance spectroscopy (EIS) at varying amplitudes. The degraded products contained in solution was identified by Fourier transform infrared spectroscopy (FT-IR). We demonstrate that the degradation degree can be monitored in real time by impedance measurements and that only degraded monomer molecules are obtained if a low potential limit is not exceeded. Finally, we showed the sensing behavior of the MIPs after degradation and biocompatibility testing according to ISO guidelines for the solution containing degraded monomer molecules. These results show that the MIPs will serve as new degraded conductive sensors for implants in the future.

## 2 Material and Methods

### 2.1 Materials

Monomer EDOT (Batch number: 483028, Purity: 97%), anion poly(natrium-4-styrenesulfonate) (Na:PSS, Batch number: 243,051) and template biotin (Batch number: 14,400, Purity: ≥99%), as well as paracetamol (Batch number: A5000, Purity: 98%) were purchased from Sigma Aldrich Chemie GmbH (Taufkirchen, Germany). Ibuprofen (Batch number: 5260, Purity: ≥99%) was purchased from Caesar & Loretz GmbH (Hilden, Germany). Platinum sheet (Purity: 99.95%) was obtained from Goodfellow GmbH (Hamburg, Germany). EIS measurements were performed in phosphate-buffered saline (PBS) solution (Batch number: 10,010,023, pH: 7.4, Life Technologies GmbH, Darmstadt, Germany).

### 2.2 Electrochemical Setup and MIP Synthesis

For the electrochemical polymerization of MIPs and NIPs, cyclic voltammetry (CV) was performed with a Zahner elektrik IM6eX potentiostat/galvanostat (Zahner Elektrik GmbH & Co. KG, Gundelsdorf, Germany). All electrochemical procedures were carried out with a three-electrode system comprising a platinum sheet (4 mm × 0.6 mm) as the working electrode, platinum wire (Ø = 0.5 mm and length = 5 mm, purity: 99.95%, Polymet-Reine Metalle. e.K., Lüneburg, Germany) as the counter electrode, and an Ag/AgCl electrode (sat. KCl) serving as the reference electrode. PEDOT:PSS was electrodeposited using CV within a potential range from −0.2 to +1.35 V, at a scan rate of 50 mV/s and 20 cycles. The measurement setup for the preparation of MIPs consisted of a three-electrode configuration in 20 mL of deionized water, 0.01 mM Na:PSS, and 30 mM EDOT. The template biotin was used at different concentrations: 3, 3.75, 5, and 7.5 mM (biotin/EDOT molar ratio: 1:10, 1:8, 1:6, and 1:4). NIPs were also electropolymerized using a similar method on the platinum electrode without adding biotin and served as the reference material. The quality of the MIP and NIP layers, which were evaluated for homogeneity and layer defects, was examined using light microscopy (Stemi 2000-C, Carl Zeiss AG, Oberkochen, Germany) and scanning electron microscopy (SEM, Zeiss Crossbeam 540, Zeiss, Oberkochen, Germany) at 5 kV and a working distance of 8 mm. A total thickness of 2.17 ± 0.4 μm was determined by SEM.

### 2.3 Electrochemical Analysis

After the deposition, the MIP and NIP sensing behavior was tested in the electrochemical analysis. MIPs and NIPs were washed in 0.1 mM sulfuric acid (H_2_SO_4_, Th. Geyer GmbH & Co. KG, Renningen, Germany), 0.1 mM sodium hydroxide (NaOH, Carl Roth GmbH + Co. KG, Karlsruhe, Germany), and 20 mL of deionized water for 20 min to remove biotin from the MIP layers. Before starting an EIS measurement, the electrodes were left in PBS for 15 min to reach an equilibrium state. This was conducted before each sample measurement. EIS was selected because impedance measurements have been used by many authors and as it represents a standard method for analyzing MIPs.^10,39,47^ In addition, impedance spectroscopy provides the best insights into the sensing processes compared to other common methods e.g., cyclic voltammetry. EIS measurements were performed using the same electrochemical setup as described in Section 2.2, with an offset of +0.15 V and an amplitude of 10 mV. The frequency range was set between 1 Hz and 100 kHz with 10 steps per decade (below 66 Hz: 4 steps per decade). A sweep mode was employed, wherein all frequencies except 100 kHz were measured twice, resulting in a total of 87 frequencies. The measurements were conducted in 20 mL of PBS with and without 4 mM biotin. The biotin concentration was selected since the range of MIP analysis in the literature (where impedance spectra of MIPs were measured in body fluid) is 1 nM to 5 mM.^10,48−50^ In addition, only a few detectable signals are expected after degradation. Therefore, to ensure comparability between before and after degradation, MIPs were measured at the highest biotin concentration of 4 mM, regardless of the biotin amount used in the deposition. Three MIPs were analyzed for each biotin concentration used for the synthesis. Note that the interleukin-6 concentration in body fluid is as low as 1 nM.^51^ To account for such low values and evaluate the performance of the developed sensors with the surrogate biotin, impedance measurements were conducted for MIPs (synthesized with an EDOT:biotin molar concentration of 1:6) in 20 mL of PBS solution with and without 1, 10, 100, and 1 μM biotin. In order to analyze the specificity of the MIPs, impedance measurements were also performed in a PBS solution with and without ibuprofen and paracetamol at the same concentration as biotin. Ibuprofen and paracetamol were selected because ibuprofen also exhibits a carboxyl group and paracetamol has a completely different structure to biotin. Four EIS measurements were performed for each solution. In order to avoid possible temperature effects, the temperature in the laboratory was controlled using a filter system and stabilized at 24 °C. The PBS solutions and biotin were stored at room temperature.

### 2.4 Electrochemical Degradation

To determine the amplitude dependence of the amount of degraded monomer molecules, electrochemical degradation was performed for NIPs and MIPs, which were prepared with a biotin/EDOT ratio of 1:6. Prior to electrochemical degradation, an acid–base wash (second) was performed as described in Section 2.3, as the MIPs were degraded after electrochemical analysis and the biotin reincorporated into the layer reduced the porosity of the MIPs. EIS measurements were performed over a period of one month (20 EIS measurements per electrode/day), with an offset of +0.2 V and an amplitude of 5, 10, or 50 mV. The impedance measurement was used because EIS is already included in the CI and the current are few nA to 1 μA, which is uncritical for long-term use in the body. Three MIPs and three NIPs per amplitude were degraded. Therefore, the degradation process involved nine MIPs and nine NIPs over a period of one month. The measurement on one electrode required 60 min, which with 18 electrodes resulted in a time of 18 h per day. The degradation was performed in 20 mL of PBS. NIPs and MIPs deposited electrodes were all fabricated at the same time and were stored in 20 mL of deionized water at room temperature when not used in experiments.

After electrochemical degradation, the layer quality was also examined by using light microscopy and SEM at 5 kV and a working distance of 8 mm. The acceleration voltage was reduced to 2 kV for the degraded MIPs at 10 and 50 mV. After the degradation, PBS containing dissolved monomer molecules was characterized by FT-IR-LUMOS II (Bruker Optik GmbH, Bremen, Germany) within a wavenumber range from 700 to 4000 cm^–1^ (64 scans, 4 cm^–1^). As a reference solution, a PBS solution containing 3 mM monomer EDOT was also measured using FT-IR. Three drops of the electrolyte solution were analyzed separately for each sample in ATR mode, and the identified spectra were averaged afterward. The spectra of the PBS solution measured under the same conditions was subtracted from the spectra of the solutions containing degraded monomer molecules. FT-IR was used to determine the electrical potential dependence of the degradation and the electrical potential limit, where only monomer molecules are degraded.

### 2.5 Fitting EIS Measurement Data

The EIS measurement data were fitted using Zview software (version 4.0h, Scribner, LLC, North Carolina) in order to determine the solution/layer resistor (*R*_0_), charge-transfer resistance (*R*_CT_) and Warburg impedance (*Z*_Warburg_). The electrical equivalent circuit for the electrochemical analysis was an *R*_0_ in series with the parallel circuit of a constant phase element (CPE) and the *R*_CT_ and *Z*_Warburg_ in series.^10,52^ Due to the change in *R*_CT_, capacitance, and diffusion coefficient during the electrochemical degradation, the stationarity/stability of the EIS is not achieved. Fitting the EIS data is difficult or impossible due to the nonstationary errors. Assuming that each EIS measurement was measured on a new system, the *R*_CT_ can be estimated using the equivalent circuit described above without *Z*_Warburg_ in a limited frequency range. The measured EIS data can be accepted to a certain extent as valid in the classical sense of EIS measurement, but further adjustment of the equivalent circuit was not allowed.^27^ The equivalent circuits are shown in the Supporting Information (Figure S1).

### 2.6 Biocompatibility

Since the MIPs are intended to be deposited and degraded subsequently on a CI electrode, a biocompatibility test of the polymers and the solution with the dissolved monomer molecule is necessary. For sample preparation, the NIPs were extracted following the surface to volume ratio indicated in ISO 10993–12:2021.^53^ Experiments were performed following ISO 10993–5:2014 guidance and were performed in biological triplicates.^54^ Extraction was performed in 500 μL of L929 Gibco RPMI 1640 cell culture media supplemented with l-glutamine and 20 mM HEPES, 10% FBS serum (PAN Good Biotech), and 5 μg/mL gentamicin for 24 h in a 24-well cell culture plate at 50 rpm rotation (Kleinschüttler KM CO_2_, Edmund Bühler GmbH, Bodelshausen, Germany) at 37 °C, saturated humidity, and 5% CO_2_ (Heraeus BBD 6220, Thermo Scientific, Waltham, Massachusetts).

Prior to the cell treatment, 5 × 104 L929 cells (mouse fibroblast cell line) were seeded into a 24-well plate and incubated (37 °C, saturated H_2_O, 5% CO_2_) for 24 h. Thereafter, cells were treated with the extracts without dilution. A blind extraction, without the test material, served as a negative control. As a positive control, the final 1% (v/v) Triton X-100 was added 5 min prior to the LDH assay. Degradation products in PBS were diluted 1:1 with 500 μL of L929 cell culture medium. For the negative and positive control, the unmodified PBS treated cells were lysed with a final 1% (v/v) Triton X-100 as required.

After the cell treatment, 50 μL of the supernatant was transferred into a 96-well culture plate in three technical replicates per biological replicate and 50 μL of lactate dehydrogenase (LDH) reagent (Cytotoxicity Detection Kit [LDH] Roche, Basel, Switzerland, prepared according to the manufacturer’s instructions) was added. After 30 min of incubation in darkness at room temperature, the absorption was measured utilizing the SpectraMax 340PC plate reader (Molecular Devices, LLC, San Jose; λ_absorption_ = 490 nm, λ_reference_ = 630 nm).

In parallel to the LDH test, the medium was replaced with 1 mL of L929 cell culture medium containing 10% of Cell Proliferation Reagent (WST-1, Roche, Basel, Switzerland). Cells were incubated at 37 °C, saturated H_2_O, and 5% CO_2_ atmosphere for 1 h. For each biological replicate, 100 μL were transferred in triplicates to a 96-well plate and absorption was measured utilizing the SpectraMax 340PC plate reader (λ_absorption_ = 450 nm, λ_reference_ = 630 nm). For the cell counting, the cells were washed twice with 0.5 mL of preheated PBS. Subsequently, cells were incubated with 0.5 mL of preheated 0.05% trypsin/EDTA for 3 min at 37 °C. The reaction was stopped by adding 0.5 mL of the L929 cell culture medium. Finally, the cell numbers were calculated utilizing the CASY Cell Counter (OMNI Life Science GmbH & Co KG, Bremen, Germany) following the manufacturer’s protocol. The cell size boundaries were set to 10 and 30 μm.

## 3 Results

### 3.1 Electrochemical Analysis

**Figure 1:**
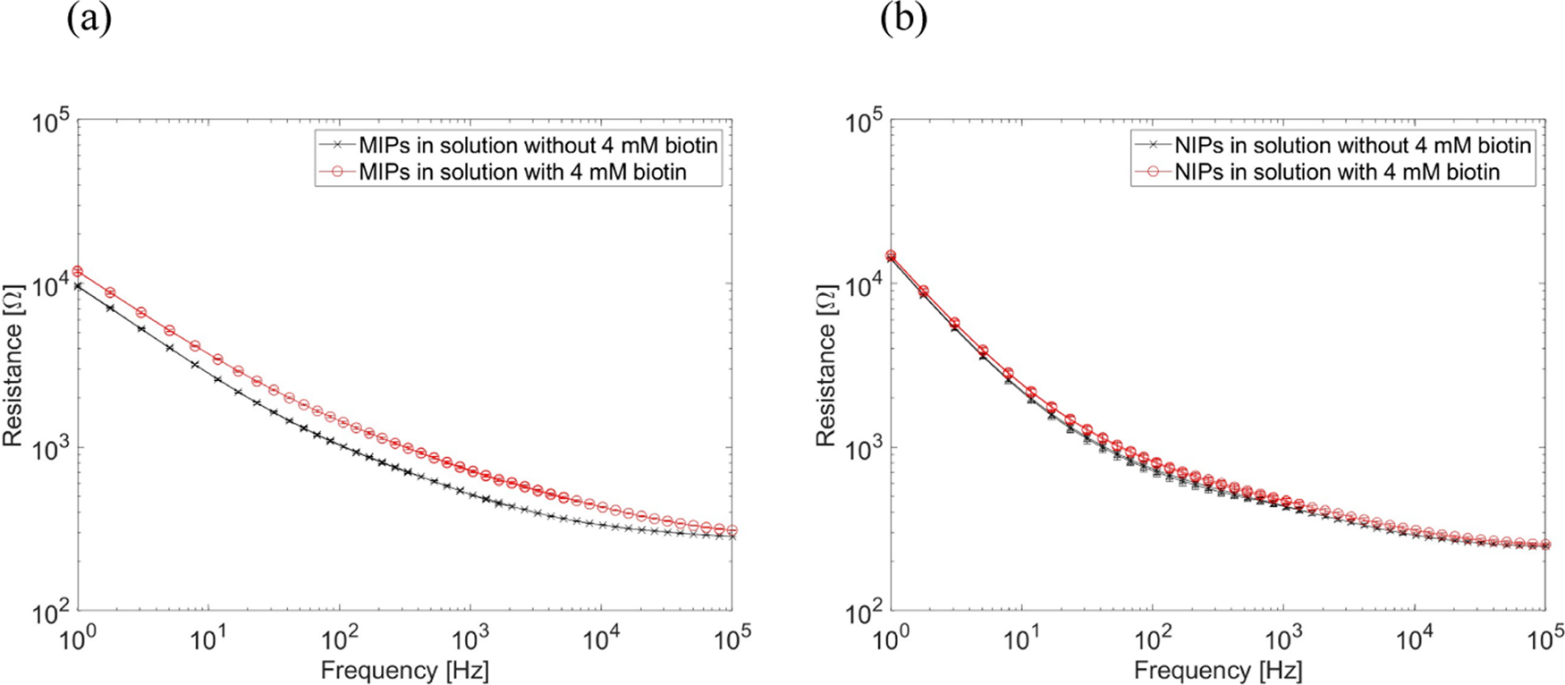
Bode plot of (a) MIPs (which were deposited with a biotin/EDOT molar ratio of 1:4) and (b) NIPs (cross symbol: EIS measurement in solution without biotin; circle symbol: EIS measurement in solution with 4 mM biotin; solid line: fit curves based on the equivalent circuit). Presented data are averaged over four EIS cycles performed on MIPs and NIPs.

The deposited MIP and NIP layers were tested for biotin sensitivity in the electrochemical analysis by EIS. It was observed from the Bode plot in Figure 1a that the impedance increased in the case of MIP electrodes in the presence of biotin at all frequency ranges. On the other hand, negligible change was observed in the case of NIP electrodes (Figure 1b). The impedance curves were fitted with the parameters determined in Table 1, and a clearly increased MIP charge-transfer resistance (*R*_CT_) was obtained in the presence of 4 mM biotin and negligible change in *R*_CT_ was observed in case of NIP electrodes. The change in *R*_CT_ indicated that biotin was successfully incorporated into the MIP layers. Moreover, a change in the solution-layer resistance (*R*_0_) was measurable. Although the differences in *R*_0_ were slight, the difference in MIPs was larger than that of NIPs, which indicated the change in conductivity of the MIP layers.

**Table 1:** Determined Fitting Parameters χ^2^, *R*_0_, and *R*_ct_ for MIPs (Which Was Deposited with a Biotin/EDOT Molar Ratio of 1:4) and NIPs in Solution with and without 4 mM Biotin^a^

	χ^2^ (×10^–5^)	*R*_0_ [Ω]	*R*_ct_ [Ω]
MIPs in solution without biotin	15 ± 7	212 ± 20	3160 ± 95
MIPs in solution with biotin	11 ± 2	194 ± 24	5868 ± 397
NIPs in solution without biotin	60 ± 40	231 ± 3	414 ± 76
NIPs in solution with biotin	38 ± 30	224 ± 6	327 ± 111

a The presented data show the mean values of three MIPs and three NIPs. Four EIS measurements per electrode were performed.

**Figure 2:**
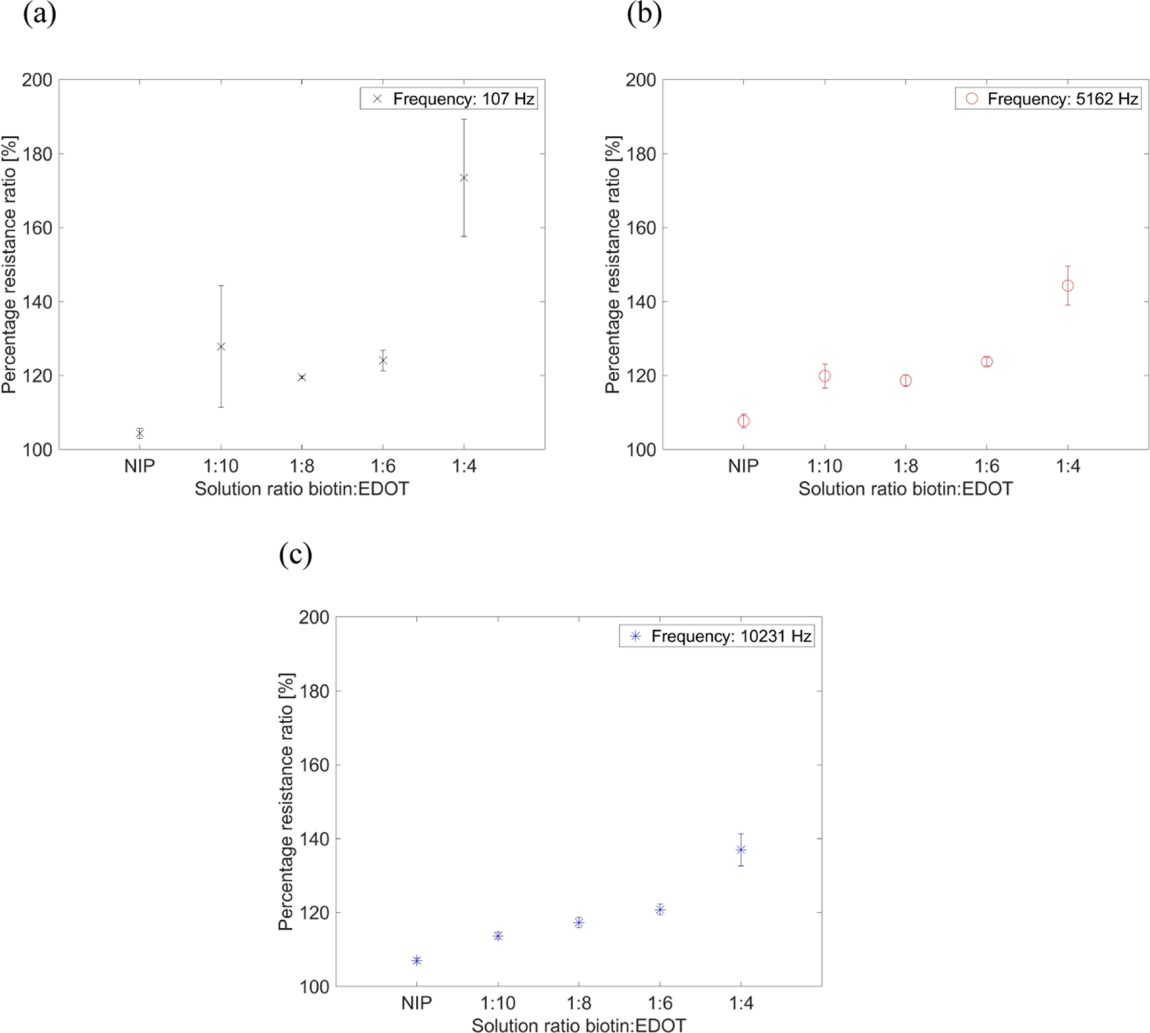
Electrochemical analysis for different biotin/EDOT ratios at frequencies of (a) 107, (b) 5162, and (c) 10231 Hz. The percentage resistance ratio is the ratio of the EIS mean value (three electrodes) of the solution with biotin and without biotin. Four EIS measurements per electrode were performed.

The biotin/EDOT molar ratio was varied in the electrochemical polymerization as follows: 1:10, 1:8, 1:6, and 1:4. It was found that the higher the ratios, the more biotin imprint was formed in the polymer layer, resulting in a greater percentage impedance change and increased layer porosity (Figure 2). In addition, the change in impedance decreased when the impedance approached *R*_0_ at higher frequencies. Moreover, at a high frequency of 10 kHz, biotin detection was still observed (Figure 2c). MIPs prepared at a 1:4 ratio had the highest deviation due to the layer porosity.

**Figure 3:**
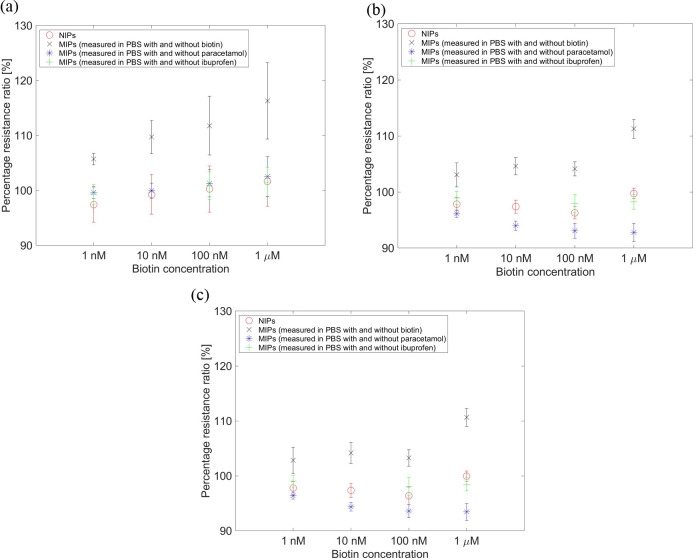
Electrochemical analysis for different biotin, paracetamol, and ibuprofen concentrations at frequencies (a) 107, (b) 5162, and (c) 10231 Hz. The percentage resistance ratio is the ratio of the mean value (three electrodes) of the solution with and without molecules (biotin, paracetamol, or ibuprofen). Four EIS measurements per electrode were performed.

Since the interleukin-6 concentration in the body fluid is approximately 1 nM, the measured concentration was reduced from 4 mM to a range of 1 nM–1 μM, as shown in Figure 3. A dependence on the biotin concentration was observed at all frequencies, but the change in impedance was small. At higher frequencies, the change was negligible at concentrations <1 μM. To analyze the specificity of the sensor, on the other hand, the MIPs were measured in a PBS solution with and without paracetamol and ibuprofen. As shown in Figure 3, no change was observed in the impedance spectra.

**Figure 4:**
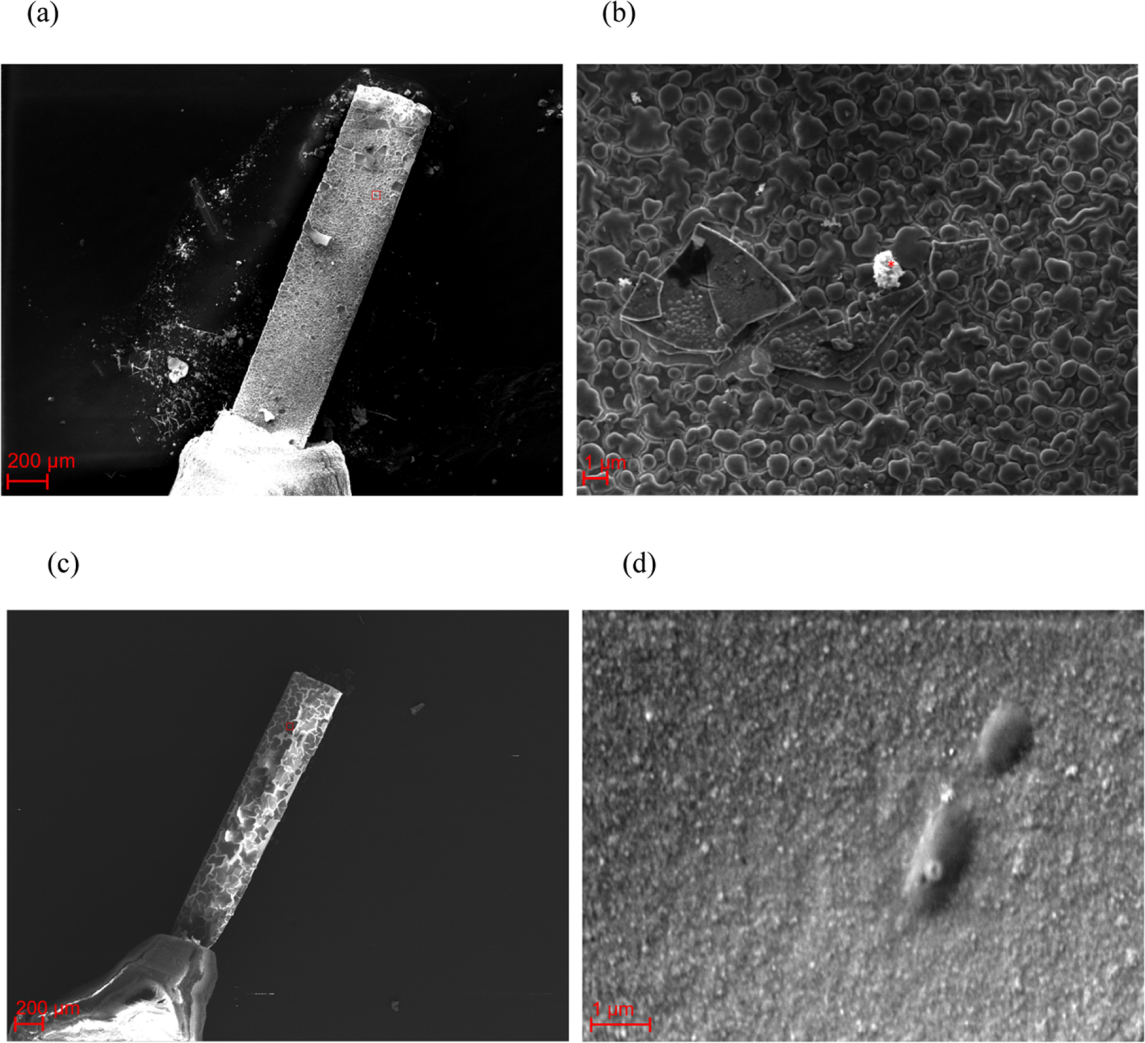
SEM images of the (a, b) MIPs (* dust particle) and (c, d) NIPs before electrochemical degradation. The rectangles in (a, c) indicated the area of (b, d).

SEM images of NIPs and MIPs before electrochemical degradation are shown in Figure 4, demonstrating that the MIP surface was more porous than the NIP surface. Moreover, both electrodes showed cracks, but this was more pronounced for the NIPs.

### 3.2 Electrochemical Degradation

Subsequently, the electrodes were electrochemically degraded by EIS with different amplitudes (degradation amplitude). An increase in *R*_CT_ in the Nyquist plot was observed as seen in the Supporting Information (Figure S2). Furthermore, the diffusion range decreased with an increasing number of EIS measurements for the NIP electrodes (Nyquist plot). All layers showed more defects and appeared to be thinner under optical microscopy after electrochemical degradation, as demonstrated in the Supporting Information (Figure S3). However, overoxidation (recognizable by a color change of the polymer layer) was not observed in any of the degraded layers. For the MIP layers (degradation amplitude of 50 mV), the polymer was detached from the electrode, and a large detached layer was observed in the PBS solution after 40th EIS measurements. A change in impedance between the first and 39th measurements was not observable. This was not the case for the MIPs with a degradation amplitude of 5 and 10 mV as well as for the NIPs.

Percentage changes in the estimated *R*_CT_ per 100 EIS measurements are shown in Table 2. The *R*_CT_ of the MIPs was very small up to the 100th EIS measurement, making fitting the EIS data difficult due to software limitations. The greater the change in *R*_CT_, the lower the impedance number and the greater the amplitude. Moreover, the change in MIPs *R*_CT_ was higher compared to that of NIPs. In addition, NIP layers were not detached from the base electrode after the 40th EIS measurement (degradation amplitude of 50 mV), as was the case with MIPs. A change (13.37 ± 5.8%) was also observed when the NIP electrodes were stored in water only. However, the change was very small compared to the applied amplitude for the first 100 EIS measurements.

**Table 2:** Percentage Change in Estimated *R*_CT_ (Average Values of Three Electrodes) per 100 EIS Measurements for Electrochemical Degradation

	NIPs			MIPs	
	5 mV [%]	10 mV [%]	50 mV [%]	5 mV [%]	10 mV [%]
%-change after second 100 EIS measurements	41 ± 16	54 ± 16	316 ± 13	28 ± 7	60 ± 8
%-change after third 100 EIS measurements	33 ± 7	36 ± 19	26 ± 6	69 ± 9	45 ± 9
%-change after fourth 100 EIS measurements	23 ± 8	34 ± 8	25 ± 9	27 ± 11	38 ± 4
%-change after fifth 100 EIS measurements	9 ± 1	15 ± 7	162 ± 12	19 ± 3	25 ± 8

**Figure 5:**
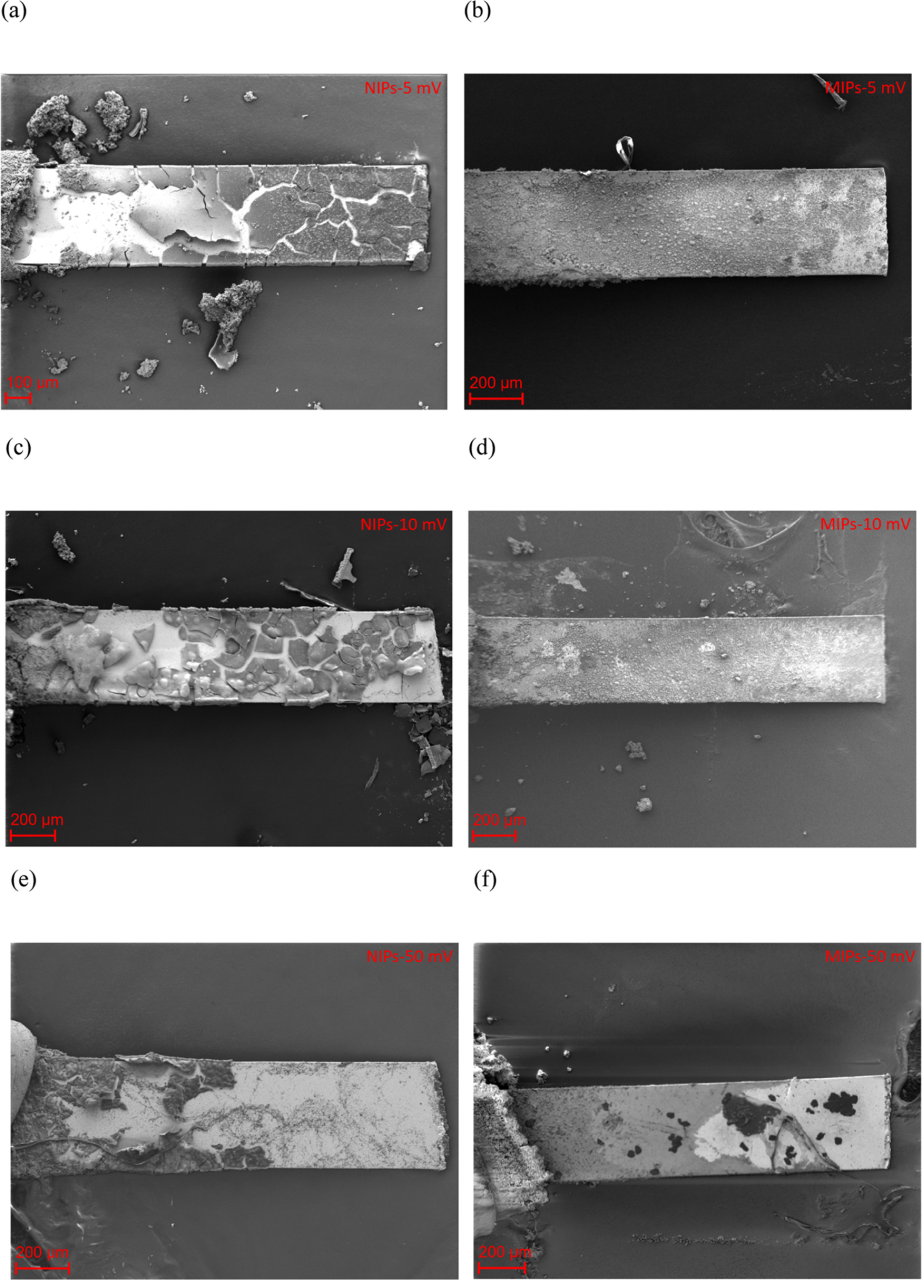
SEM images after electrochemical degradation for the NIPs at amplitudes of (a) 5, (c) 10, and (e) 50 mV, as well as for the MIPs at amplitudes of (b) 5, (d) 10, and (f) 50 mV.

SEM images after electrochemical degradation are shown in Figure 5. The NIP layers increasingly degraded as the amplitude increased. For the MIPs, on the other hand, a thin layer was observed at all amplitudes. At amplitudes of 10 and 50 mV, the SEM acceleration voltage had to be reduced, which decreased the analysis depth. This indicated that the layer thicknesses of the MIPs at a degradation amplitude of 10 and 50 mV were thinner than the MIPs with an amplitude of 5 mV and the NIP layers. Although the MIP layers (degradation amplitude of 50 mV) were not observed under the optical microscope, a very thin layer was observed by SEM, which may be the first/second deposited polymer molecular layer. This showed that the MIP layers with an amplitude of 50 mV were thinner than the other MIP and NIP layers. Moreover, pronounced cracks were observed in the NIPs whereas the MIPs only exhibited small cracks.

**Figure 6:**
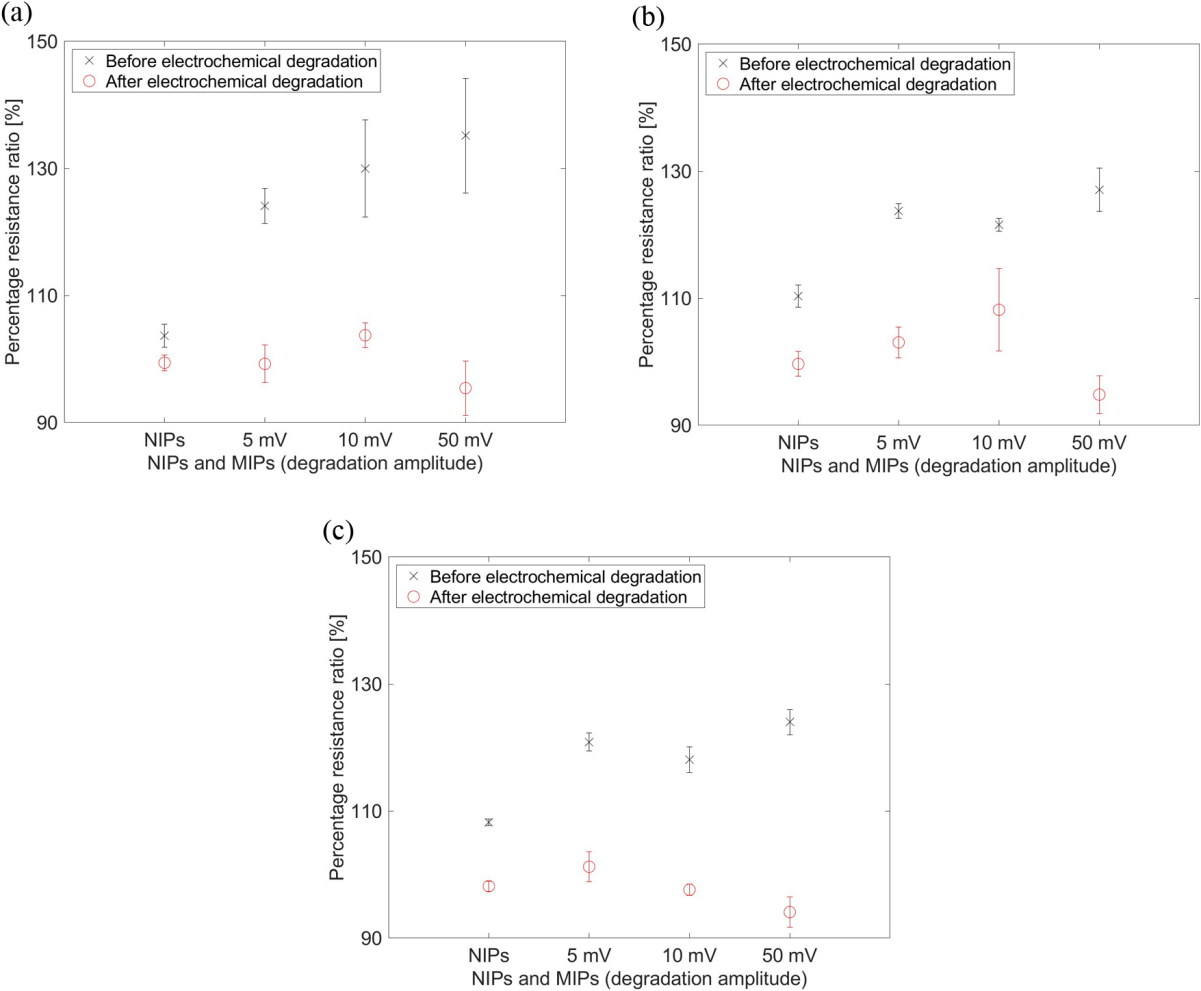
Electrochemical analysis before and after electrochemical degradation at frequencies of (a) 107, (b) 5162, and (c) 10,231 Hz (cross symbol: before electrochemical degradation, circle symbol: after electrochemical degradation).

The electrochemical analysis was also performed after degradation, as shown in Figure 6. MIPs had a negligible biotin detection capacity after degradation. Although the MIPs were still present in the form of a thin layer after the degradation, the biotin imprint in the MIPs was lower after degradation.

**Figure 7:**
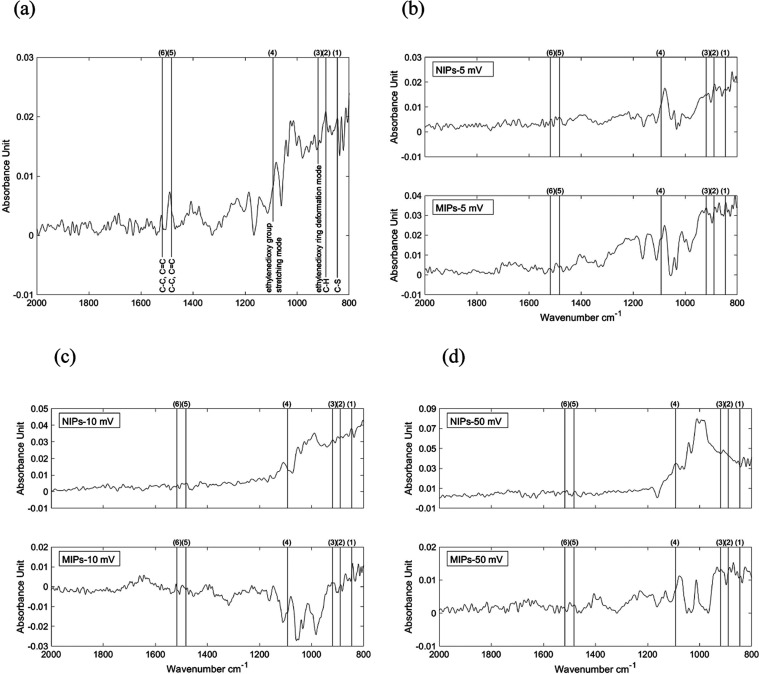
FT-IR measurement of the (a) monomer-containing reference solution. In addition, the FT-IR spectra of the solution in which the polymers were degraded with amplitudes of (b) 5, (c) 10, and (d) 50 mV (NIPs: upper diagram and MIPs: bottom diagram). The vertical lines mark the determined bindings ((1) C–S bond: 846 cm^–1^, (2) C–H bond: 890 cm^–1^, (3) ethylenedioxy ring deformation mode: 920 cm^–1^, (4) stretching modes of the ethylenedioxy group: 1093 cm^–1^, C–C and C=C bonds: (5) 1483 cm^–1^ and (6) 1518 cm^–1^).

After electrochemical degradation, the solutions with dissolved monomer molecules were analyzed by FT-IR. The C–S bond at 846 cm^–1^, C–C and C=C bonds at 1483 and 1518 cm^–1^ were determined for all solutions, as seen in Figure 7. The C–H bond at 890 cm^–1^, on the other hand, was present in all solutions except for the electrolyte solution of NIPs (degradation amplitude of 50 mV). A slight shift from the C–H bond was determined, where the shift increased as the amplitudes increased, as demonstrated in Table 3. In addition, the band at 920 cm^–1^, which is assigned to the ethylenedioxy ring deformation mode, was determined for all solutions but not for the NIP solution (degradation amplitude of 5 mV). The band at 1093 cm^–1^ ascribed to the stretching modes of the ethylenedioxy group was observed for the NIP (degradation amplitude of 50 mV) and MIP solutions but not for the NIP solutions with a degradation amplitude of 5 and 10 mV.

**Table 3:** Wavenumber Peak of the C–H Bond in a Solution of the Degraded NIPs and MIPs

	NIPs		MIPs		
	5 mV [cm^–1^]	10 mV [cm^–1^]	5 mV [cm^–1^]	10 mV [cm^–1^]	50 mV [cm^–1^]
C–H bond (890 cm^–1^)	888	886	887	886	884

### 3.3 Biocompatibility Test

**Figure 8:**
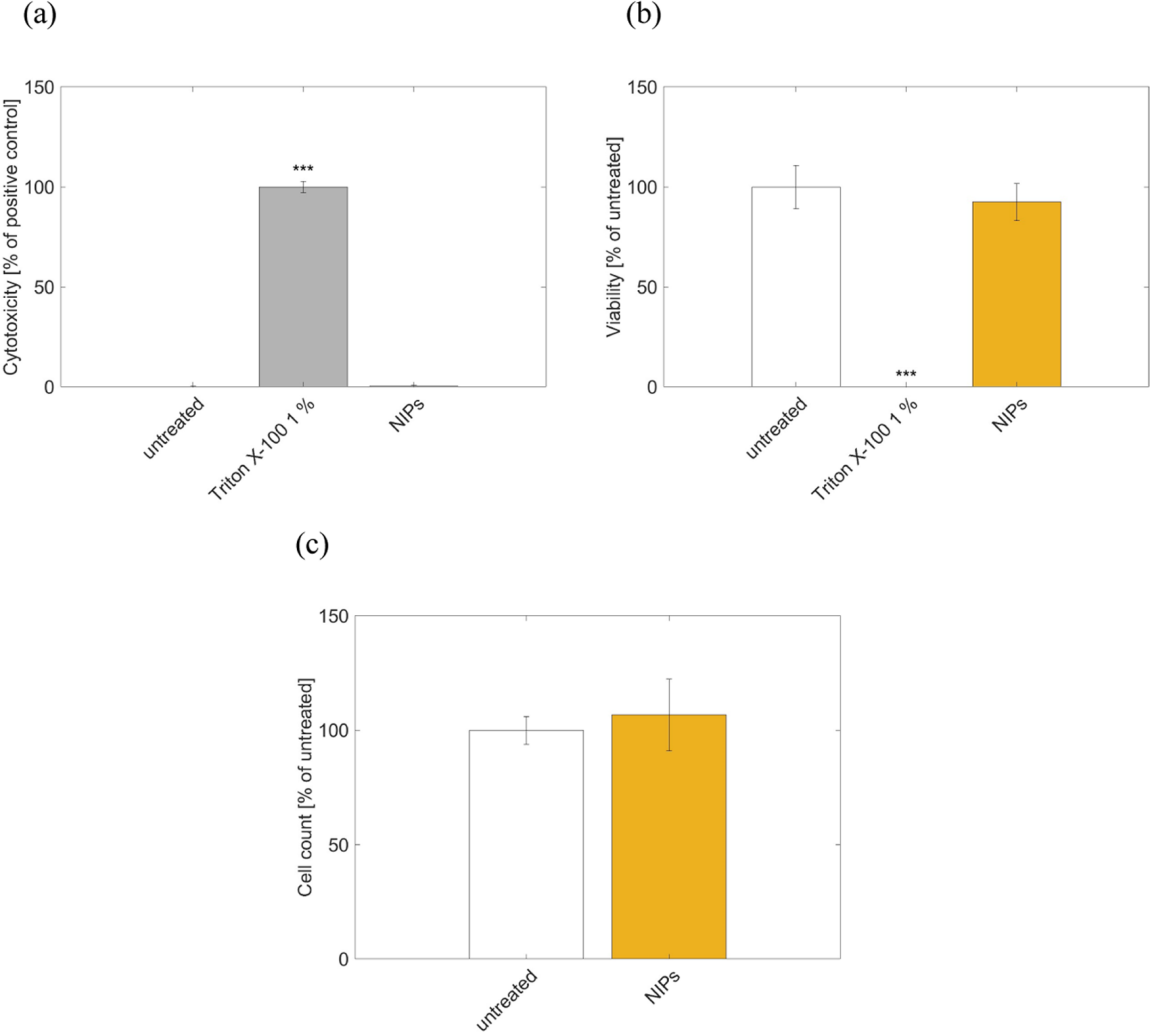
Biocompatibility assessment of NIPs following ISO guidance. Cytotoxicity of NIP extract was assessed utilizing (a) lactate dehydrogenase (LDH), (b) water-soluble tetrazolium salt (WST-1), and (c) cell counting. Measurement was performed after 24 h of extraction and 24 h of treatment. As model system served L292 cells. Data represent means ± SD of biological triplicates. Statistically significantly different results from neg. control (untreated cells): *** *p* < 0.001, one-way ANOVA with Dunnett follow-up.

In terms of biocompatibility, the cytotoxicity assessment following ISO 10993–5 and ISO 10993–12 guidance utilizing L929 cells is depicted in Figure 8. Extracts of the NIP polymer did not cause cell membrane damage (LDH), did not impair metabolic competence (WST-1), and did not reduce the cell count.

**Figure 9:**
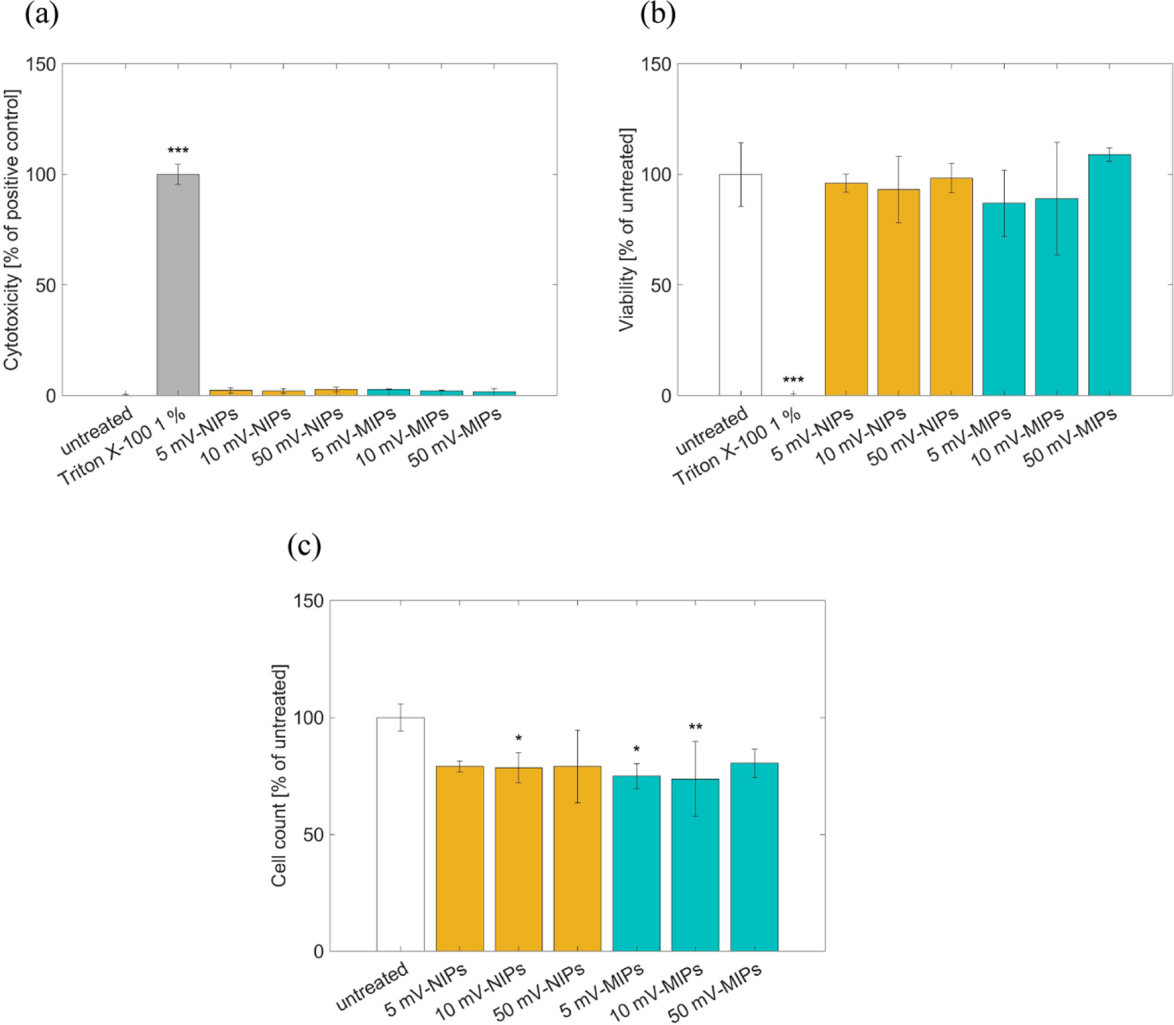
Evaluation of polymer degradation. Cytotoxicity of degradation products of NIP and MIP polymers in PBS were scrutinized utilizing (a) lactate dehydrogenase (LDH), (b) water-soluble tetrazolium salt (WST-1), and (c) cell counting. Measurement was performed after 24 h of extraction and 24 h of treatment. As model system served L292 cells. Data represent means ± SD of biological triplicates. Statistically significantly different results from neg. control (untreated cells): * *p* 0 < 0.05, ** *p* < 0.01, one-way ANOVA with Dunnett follow-up.

Degraded polymers, akin to the NIPs, did not impair the membrane integrity (LDH), as shown in Figure 9a. Nonetheless, a nonsignificant reduction in metabolic competence was observed after incubation with degradation products, as demonstrated in Figure 9b. The cell count, as the most sensitive cytotoxicity measure applied here, was reduced in a significant manner for NIPs to 78 ± 6% viable cells and MIPs to 74 ± 16% viable cells at 10 mV and for MIPs also at 5 mV to 75 ± 5% viable cells, as shown in Figure 9c. For the other solutions, the reduction of the cell count was not observed.

## 4 Discussion

### 4.1 Electrochemical Analysis

In this study, MIP and NIP layers for biotin sensing, as an initial replacement for interleukin-6, were produced by using electrode deposition. When EIS measurements were performed, the *R*_CT_ change of the MIPs indicated that binding of biotin to the polymer surface or blocking the absorption site increases the energy for electron transfer, leading to the successful incorporation of biotin into the MIP layers. This conclusion has also been reached by many authors who have produced MIPs with a different target molecule and measured by EIS.^8,10,39,52^ The change in resistance *R*_0_ showed that MIPs exhibit enhanced conductivity due to the probable incorporation of negatively charged biotin. As MIP synthesis took place in water at a pH value of 7, biotin (isoelectric point of 3.5) had either a carboxy group or a negatively charged carboxylate group.^55^ PEDOT:PSS is positively charged, making the probability of hydrogen bonding between the polymer and biotin with a carboxylate group more likely. This type of bonding was also concluded by other authors who used another negatively charged template.^10,39,52^ The incorporation of negatively charged biotin reduced the binding sites for PSS. In addition, the polymer layer showed cracks due to the PSS’s hydrophilicity, as seen in the SEM images. Contact with air leads to the contraction of polymers, which results in cracks. Since there is more PSS in the NIPs, the cracks were more pronounced. Moreover, when biotin is dissolved by washing, the polarons/bipolarons become unstable, which reduces the electrochemical activity and increases the porosity of the MIP layer. By reintegrating biotin, the polarons/bipolarons are stabilized, and electrochemical activity increased, which explained the slight decrease in the *R*_0_ value of MIPs. Although the change in NIPs *R*_CT_ was negligible, the change was caused by nonspecific adsorption of biotin on the NIP surface. Therefore, the NIPs can be used to determine nonspecific adsorption or adhesion on the surface. Since the applications of MIPs on the CI electrode are performed later *in vivo*, adhesion of certain proteins is possible, which leads to a false positive result or a reduction in the *R*_CT_ change. Wackers et al. used this approach, in which the MIP data were adjusted for electrode fouling by the difference between MIPs and NIPs.^10^ In addition, pulses that are already implemented in the CI can counteract the adhesion of certain proteins. As these pulses last a few μs, the possibility of the MIPs and NIPs being affected during this period is negligible.

The biotin/EDOT ratio was varied for the MIP deposition. It was obvious that more biotin was incorporated at higher ratios, resulting in more porous MIP layers. During the acid–base wash, part of the MIP layer detached from the electrode due to its inherent porosity, resulting in a reduced detection area and a high standard deviation of the MIPs, which were prepared in a ratio of 1:4.

The measured concentration of biotin was reduced to 1 nM–1 μM, as the interleukin-6 concentration is 1 nM in the body fluid. At higher frequencies of 5162 and 10,231 Hz, negligible impedance change was measured at concentrations <1 μM, as the impedance approaches the solution resistance. On the other hand, at higher biotin concentrations much more biotins are incorporated into the polymer during the impedance measurement, resulting in a larger change at higher frequencies. The sensitivity of the sensor is at a biotin concentration of 1 μM, as an impedance change was measured at frequencies of 5162 and 10,231 Hz. At lower frequencies, on the other hand, impedance change was measured at all concentrations. However, the change at 1 nM is very small or negligible. Therefore, the sensitivity of the MIPs is at a minimum concentration of 10 nM. A dependence on concentration has also been observed in the literature; Wackers et al. detected a change in impedance at a concentration of 1 nM. This may be due to the fact that more imprinting was incorporated or the layer thickness was much larger. The target molecules can diffuse into the polymer layer and dock to the binding sites in the lower polymer layer, whereby a greater change can be achieved. Since diffusion into the polymer layer is restricted, change, however, is limited. Further, one possible solution to increase the impedance change at lower measurement concentrations is to electrically connect several MIPs together. This was also conducted by Wackers et al. where a better signal was obtained.^10^

In addition, the MIPs were also measured in PBS with paracetamol and ibuprofen. Although ibuprofen also has a carboxylate group, no impedance change was detected. This was also the case with paracetamol. Therefore, both paracetamol and ibuprofen were not incorporated into the polymer during the impedance measurement. This indicates that the MIPs are specialized for biotin.

Compared with biotin, interleukin-6 consists of amino acid sequences. The amino acid, similar to biotin, has a carboxyl group, which becomes a carboxylate group when the pH value is increased. Since glutamic acid (isoelectric point: 3.08) and aspartic acid (isoelectric point: 2.98) belong to the amino acid sequences of interleukin-6 and have a similar isoelectric point as biotin (isoelectric point of 3.5), the hydrogen bond between the polymer and interleukin-6 via this amino acid is very likely.^55,56^ This conclusion assumes that the pH value during the deposition of MIPs is 7. Nevertheless, since the molecular weight of interleukin-6 is larger than that of biotin, it can be assumed that the MIPs become much more porous with the incorporation of interleukin-6.

Further, since the applications of the MIPs on CI electrodes are performed later *in vivo* (body temperature: 37 °C), the increase in temperature can influence the detection phase. The diffusion can accelerate the molecular detection and slightly decrease the impedance.^10^ On the other hand, the conformation of biotin and interleukin-6 is not changed, resulting in no change in sensitivity. As an Arrhenius evaluation still delivers values close to 1, it can be assumed that the effect of temperatures from 24 to 37 °C varies in the range of secondary approximation. In addition, the temperature during the study was always controlled and kept constant near 24 °C by the filter system. These results prove that the deposited layers consisted of MIPs.

### 4.2 Electrochemical Degradation

In the degradation experiment, as the *R*_CT_ increased the thinner and more defective the polymer layer became. The more polymer defects that occurred, the more the platinum surface was in contact with electrolyte solution, whereby the impedance of the platinum affected the impedance of the system more and thus led to an *R*_CT_ change. This was also the reason why the changes became smaller with the number of EIS measurements, as the layers got increasingly thinner. In addition, polymers/monomer molecules with lower binding energy can be separated from the polymer during the first EIS measurement. In parallel to polymer degradation, the polymer structure can also change, increasing the *R*_CT_. However, the decreasing diffusion range during NIP degradation was also an indication of the decreasing layer thickness after degradation. Besides, the analytical depth of the MIPs with a degradation amplitude of 10 and 50 mV were reduced and the MIP layers (degradation amplitude of 50 mV) were not observed under the optical microscope, showing that the layers became thinner with higher applied amplitudes. The layer received more energy to break the chain at higher degradation amplitudes, resulting in an observable amplitude dependence. Moreover, since the deposition method was the same for the NIPs, the number of cracks/trenches was higher at 10 or 50 mV, which was interpreted as an effect of electrochemical degradation. However, no change was observed in the MIPs (degradation amplitude of 50 mV) up to the 39th EIS measurement. This may be due to the fact that the degraded polymer segment was still bound to the electrode, leaving the polymer segment on the electrode. These connections were separated in the following EIS measurements, resulting in a large observable change. This may also be the reason for the large change in the NIPs (50 mV) after the 500th EIS measurement and MIPs (5 mV) after the 300th EIS measurement and the small change after the 200th EIS measurement. Since no high currents were measured, an incorrectly applied voltage due to the mismatch of the reference electrode was neglected. Further, the results of the Nyquist diagram and the SEM analysis indicated that the MIPs were more degraded compared to NIPs. As the NIPs and MIPs were treated under the same conditions after deposition, the MIPs degraded more than NIPs due to their higher porosity. In addition, the fact that NIP layers stored in deionized water for one month had a lower change in degradation indicated that the change was amplified with the application of voltage or additional energy. Further, since the PSS is hydrophilic, the change can be caused by hydrolysis. Hydrolysis causes the polymer to separate by a reaction with water. The duration of the hydrolysis reaction depends on the pH value or morphology, among other factors, and varies from several hours to years. Since no overoxidation was observed during degradation and due to the hydrophilic nature of PSS, the EIS can accelerate hydrolysis.^35^ In addition, the change of MIPs in the liquid environment without applying voltage can be higher compared to that of NIPs due to their porosity. Since the inflammation detection phase can last up to one month and the MIPs remain in a liquid environment *in vivo*, the parameter selection of the template/monomer molar ratio is crucial. The parameter must be adjusted to ensure that the change is as negligible as that for NIPs without a large effect on sensitivity.

In the literature, the conductive polymer layer was only degraded by overoxidation with a high applied potential (≥0.8 V). The polymer layer detached from the electrode after overoxidation or after the application of the potential, and large polymer segments were also observed in the solution.^27,28,30−32,57^ The results are similar to the results with MIPs with a degradation amplitude of 50 mV (applied potential: 0.25 V) but not with NIPs and MIPs with a degradation amplitudes of 5 mV (applied potential: 0.205 V) and 10 mV (applied potential: 0.21 V). Since no overoxidation (after degradation) was observed, the degradation of MIPs and NIPs was possible with lower applied potential and without overoxidation.

MIPs had a negligible biotin detection capacity after degradation due to fewer biotin imprints in the lower layers, meaning that no great difference was observed in the impedance. Since the polymer structure can change in parallel with polymer degradation, it is possible that the biotin imprints have changed as a result, making sensing no longer possible. The other authors also concluded from their results that a change in the polymer structure is possible in addition to degradation in the case of overoxidation. However, the structural change of conductive polymers during overoxidation is currently being discussed and requires additional research. The changes explained by Barsch et al. is widely accepted for overoxidation.^27−29,57^ Since no overoxidation was observed after the degradation of NIPs and MIPs, a change in the polymer structure must also be analyzed. On the other hand, it is currently discussed in the literature that the polymer layer detaches from the electrode layer by layer during overoxidation.^27,28,57^ If this argument is also accepted for electrochemical degradation, new detection areas can be exposed in the lower polymer layer as a result of degradation. This increases the lifetime of the sensor. However, since the diffusion of the target molecules into the polymer layer can occur during the detection phase (before degradation) and bind to the lower detection area, degradation can cause the change in *R*_CT_ to decrease. Since diffusion is limited, the reduction in the change in *R*_CT_ is low.

In the FT-IR measurement, a shift of the C–H bond peak increased with increasing amplitude, demonstrating that the presence of PEDOT with α,α′-coupling increased. It is obvious that the strong band ascribed to the C–H bond at 890 cm^–1^ disappears in the polymer spectrum, as it was the case for NIP solutions (degradation amplitude of 50 mV). In addition, the ethylenedioxy ring deformation mode at 920 cm^–1^ and the stretching modes of the ethylenedioxy group at 1213 cm^–1^ were also determined for the NIP solutions (degradation amplitude of 50 mV), which are only identified for the polymer spectrum in the literature.^58,59^ For MIP solutions (degradation amplitude of 50 mV), on the other hand, the C–H bond was still observed due to layer porosity. In addition, the ethylenedioxy ring deformation mode and the stretching modes of the ethylenedioxy group were not measured for the NIP solutions (degradation amplitude of 5 mV). However, the C–H shift was still observed, indicating that negligible amounts of degraded polymer were also present in the solutions in addition to monomer molecules. These results indicate that at amplitudes of less than 5 mV, only degraded monomer molecules can be obtained that allow renal clearance. Furthermore, we have proven that degradation is possible without overoxidation and that degradation is controlled and monitored. With this degradation, we are able to regulate the number of monomer molecules and whether monomer molecules are degraded via the applied potential.

Since inflammation can occur between the first day after surgery and up to 6 weeks afterward, the MIPs should be detectable for up to one month and then degraded in a controlled and monitored manner. It is expected that the impedance measurements for inflammation detection and degradation must be performed for at least three months. As the maximum current for the impedance measurement is 1 μA (max. voltage: 205 mV), the maximum electrical wattage consumed is 0.2 μW, meaning a maximum consumption of 0.45 mWh over the course of three months of impedance measurements. The current cochlear implant batteries have a capacity of approximately 0.8 mWh, which ensures that impedance measurements can be performed for three months. Since stimulation of the CI electrode, on the other hand, is performed in parallel, battery exchange is possible during the degradation phase. As the degradation is performed by applying a voltage or by EIS, a continuation of the degradation or impedance measurement is possible after the battery exchange. Therefore, no further energy source is required for the future detection of inflammation and degradation of MIPs.

### 4.3 Biocompatibility Test

The polymer showed no cytotoxicity within the biocompatibility end point evaluation conducted in this study following the guidance of ISO 10993–5 and ISO 10993–12.^53,54^ In contrast, the degradation products impaired the metabolic competence in a nonsignificant manner, while not damaging the cell membrane (LDH). Further, the cell count was significantly reduced at 10 mV for both polymers. Therefore, it can be concluded that the degradation products hindered cell metabolism and thus reduced the cell count. Since less degraded polymers and more monomer molecules are obtained at low voltage, it can be postulated that the monomer molecules can induce molecule cytotoxic effects in L929 cells. In a real-life scenario, where the degradation will be performed in a stepwise manner, the release of these monomer molecules will take place over a long period of time (approximately one to three months). Thus, the released monomer molecules are excreted via the renal system, as stated above, and their concentration will be much lower in the inner ear as compared to this *in vitro* test setup in which the cells were treated with a worst-case scenario. In addition, as the reduction of cell viability in different voltages is found to be at similar levels, the induced cytotoxicity might be limited. Despite measuring the cytotoxicity for the degradation products, the cytotoxicity values were within the allowed range of ISO 10993–5. This document states that only cytotoxicity above 30% should be considered. As all cell count values were >70%, this was considered acceptable. The excellent biocompatibility of the PEDOT:PSS polymer used has also been confirmed by other authors.^13−15,47,60,61^ Therefore, our results in accordance with the literature show that the use of the polymer PEDOT:PSS as MIPs and their degradation are safe for implant application.

## 5 Conclusions

We have developed a novel controlled electrochemical degradation method of conductive MIPs for future application of inflammation sensing in a cochlear implant. The MIP and NIP layers based on PEDOT:PSS were deposited on a platinum electrode by electrochemical deposition. The electrochemical analysis using EIS confirmed the successful incorporation of biotin into the MIPs layer, which affected the *R*_CT_ and conductivity of the layer. The MIP and NIP layers were subsequently degraded in a controlled amplitude-dependent manner and were additionally monitored by EIS. The degraded monomer molecules were obtained only if the applied voltage of 0.205 V (offset: 0.2 V and amplitude: 5 mV) was not exceeded. The evaluation of cytotoxicity showed a nonsignificant effect on metabolic competence and cell membrane integrity, with a significant decrease in cell number at higher degradation amplitudes. The biocompatibility results adhere to the guidelines of ISO 10993.^53,54^

An important step toward the detection of inflammation was taken in this study. According to the best of our knowledge, this report is the first study of the controlled and monitored degradation of MIPs based on a conductive polymer in which monomer molecules (allowing renal clearance) are obtained. Furthermore, the degradation product and polymer are cytotoxically safe according to ISO guidelines. These results are future-oriented for MIP application on CIs and for fulfilling the strict medical approval regulations. This type of MIP-based sensor may also represent a new, better alternative to known sensors based, e.g., on nonconductive degradable polymers or copolymers, due to the advantages mentioned above that also include simple fabrication and reliable reproduction.

## Supporting Information Available

Additional experimental details, including the equivalent circuit, the Nyquist plot and optical microscopy images of MIP and NIP degradation (PDF)
