# Supplementary material for: Electrochemical Degradation of Molecularly Imprinted Polymers for Future Applications of Inflammation Sensing in Cochlear Implants
Source: ACS Omega. 2024 May 24;9(23):25223–38. doi: 10.1021/acsomega.4c02906 (PMC11170751; doi:10.1021/acsomega.4c02906)
Supplement: Supplementary file 1 — ao4c02906_si_001.pdf [file ao4c02906_si_001.pdf]

Electrochemical degradation of molecularly imprinted polymers for future applications of inflammation sensing in cochlear implants

Minh-Hai Nguyen[1], Adrian Onken[1], Jan Sündermann[2], Madina Shamsuyeva[3], Pankaj Singla[4], Tom Depuydt[5], Marloes Peeters[4], Patrick Wagner[5], Konrad Bethmann[6], Julia Körner[7],Hans-Josef Endres[3], Thomas Lenarz[1], Theodor Doll[1].*

[1]Department of Otolaryngology and Cluster of Excellence "Hearing4all", Hannover Medical School, Carl-Neuberg-Straße 1, 30625 Hannover, Germany; nguyen.minh-hai@mh-hannover.de; Onken.Adrian@mh-hannover.de; Lenarz.Thomas@mh-hannover.de; Doll.Theodor@mh-hannover.de

[2]Department of Chemical Safety and Toxicology, Fraunhofer Institute of Toxicology and Experimental Medicine ITEM, Nikolai-Fuchs-Straße 1, 30625 Hannover, Germany; jan.suendermann@item.fraunhofer.de

[3]IKK - Institute of Plastics and Circular Economy, Leibniz University Hannover, An der Universität 2, 30823 Garbsen, Germany; shamsuyeva@ikk.uni-hannover.de; endres@ikk.uni-hannover.de

[4]Engineering Department, University of Manchester, Engineering A building, Booth E Street, M13 9QS, Manchester, United Kingdom; pankaj.singla@manchester.ac.uk; marloes.peeters@manchester.ac.uk

[5]Laboratory for Soft Matter and Biophysics, KU Leuven, Celestijnenlaan 200D, Leuven B-3001, Belgium; tom.depuydt1@kuleuven.be; patrickhermann.wagner@kuleuven.be

[6]Department of Information processing, Leibniz University Hannover, Welfengarten 1, 30167 Hannover, Germany; bethmann@tnt.uni-hannover.de

[7]Institute of Electrical Engineering and Measurement Technology, Leibniz University Hannover, Appelstraße 9a, 30167 Hannover, Germany; koerner@geml.uni-hannover.de

Supporting Information SI

The impedance spectrum of the MIPs and NIPs has been modelled with the modified Randles cell, as demonstrate in Figure S1.

(a) (b)

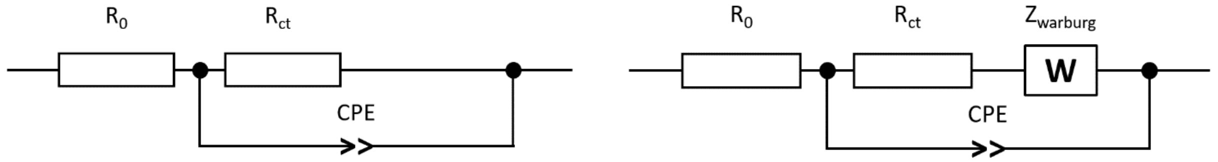

Figure S1. Electrical equivalent circuit for fitting a) the degradation impedance data and b) the electrochemical analysis impedance data.

The fitting of the model to the electrochemical degradation data are shown in Figure S2.

(a)

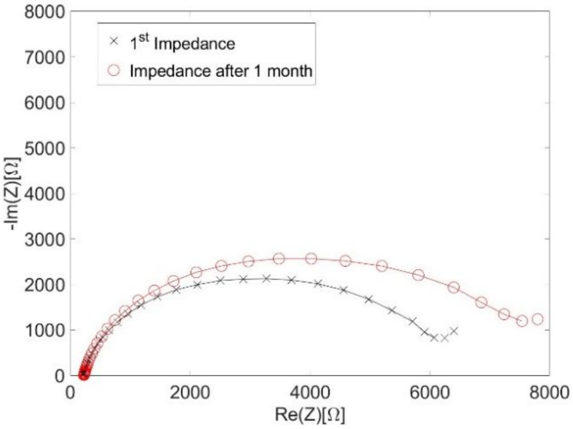

(b)

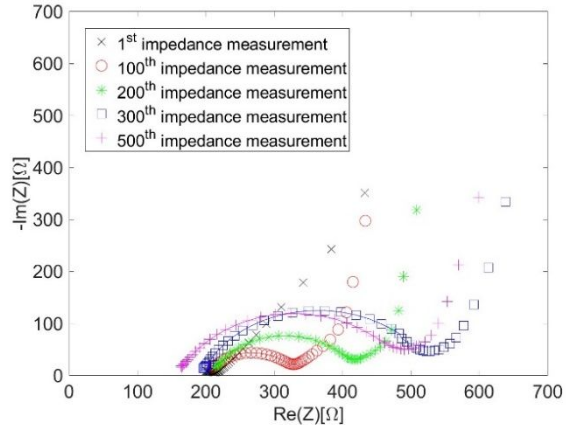

(c)

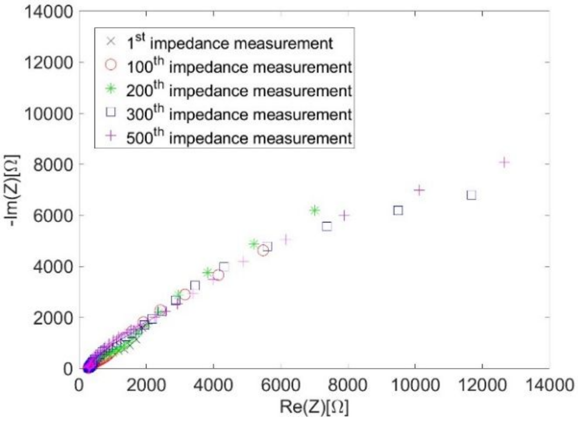

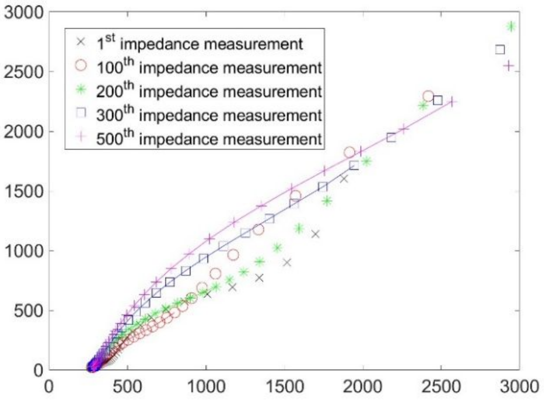
1st impedance measurement
100th impedance measurement
200th impedance measurement
300th impedance measurement
500th impedance measurement

(d)

(e)

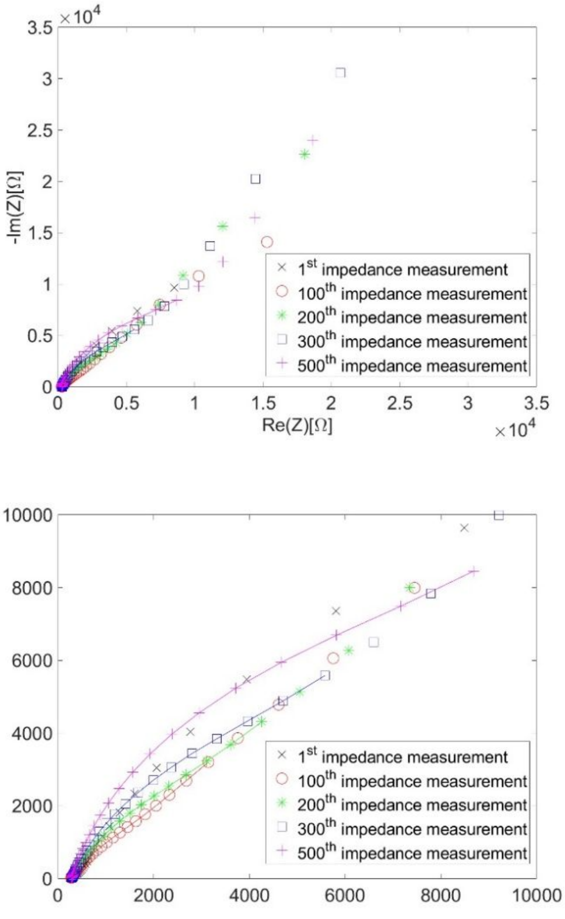
×10^4
-Im(Z)[Ω]
Re(Z)[Ω]
×10^4
1st impedance measurement
100th impedance measurement
200th impedance measurement
300th impedance measurement
500th impedance measurement
1st impedance measurement
100th impedance measurement
200th impedance measurement
300th impedance measurement
500th impedance measurement

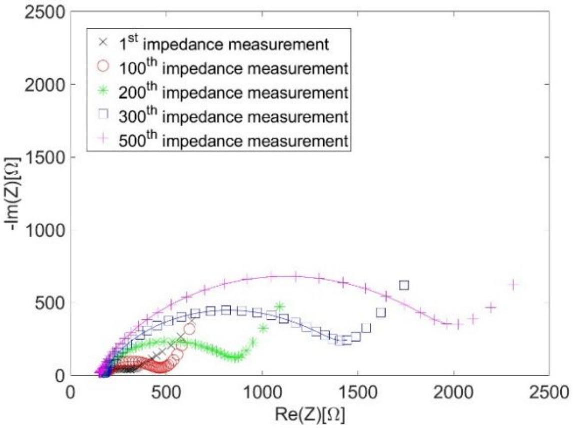
1st impedance measurement
100th impedance measurement
200th impedance measurement
300th impedance measurement
500th impedance measurement
-Im(Z)[Ω]
Re(Z)[Ω]

(f)

(g)

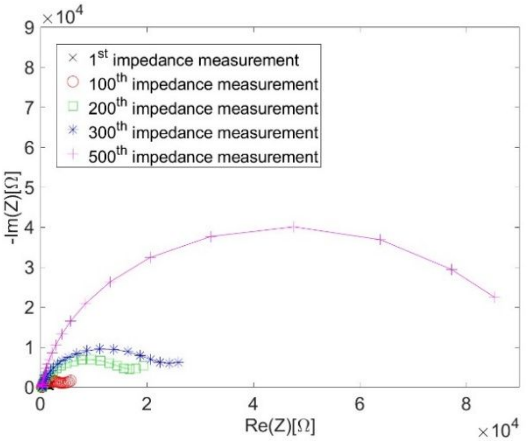

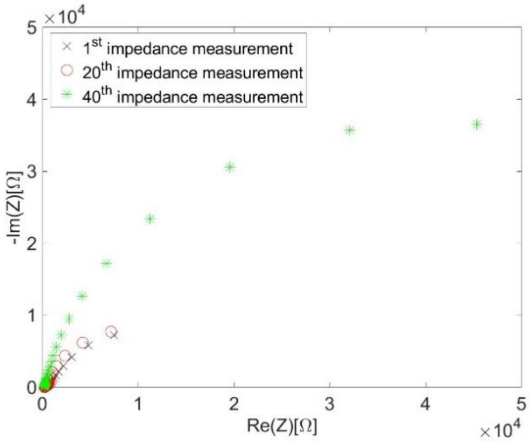

Figure S2. Impedance data for the electrical degradation. NIP, which was a) only stored in water for one month. NIP, which was degraded at an amplitude of b) 5 mV, d) 10 mV and f) 50 mV. MIP degraded at an amplitude of c) 5 mV, e) 10 mV and g) 50 mV.

The optical microscope imagines before and after the electrochemical degradation are shown in Figure S3.

(a)

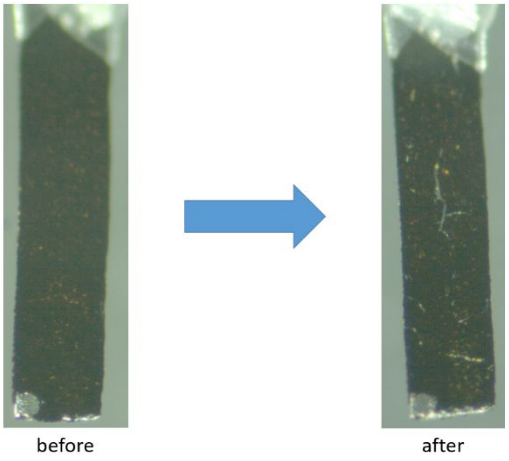

(b)

(c)

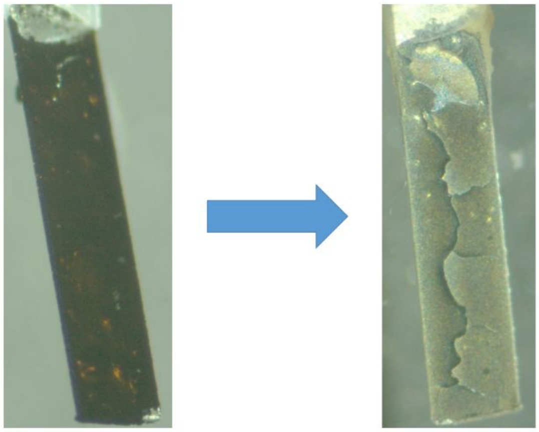
before
after

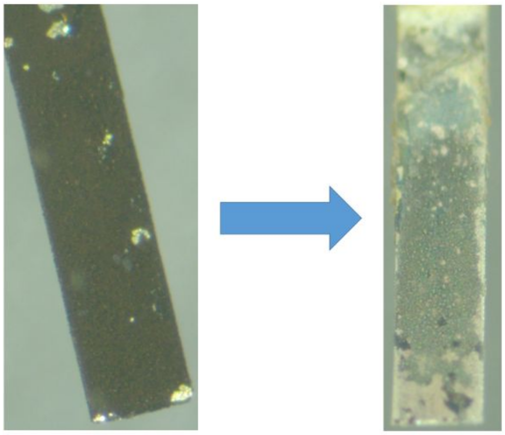
before
after

(d)

(e)

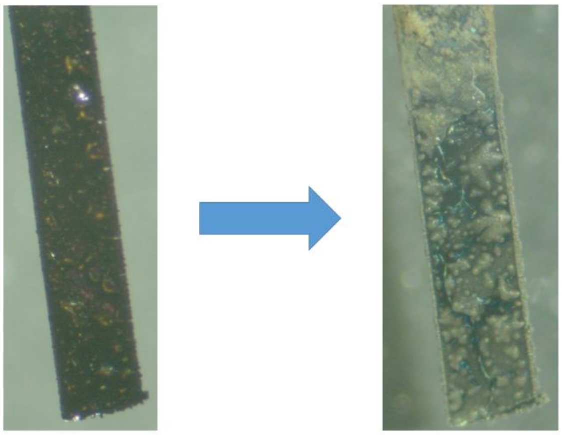
before
after

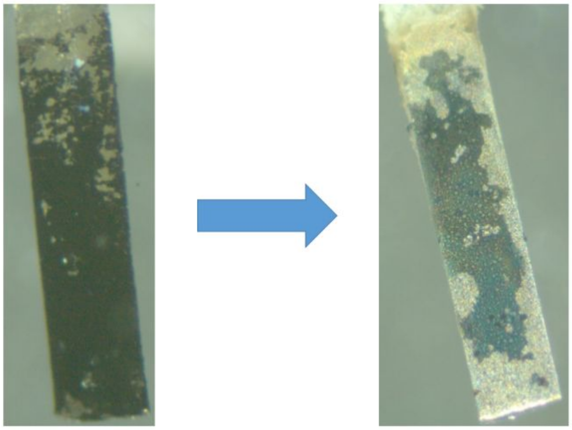
before
after

(f)

(g)

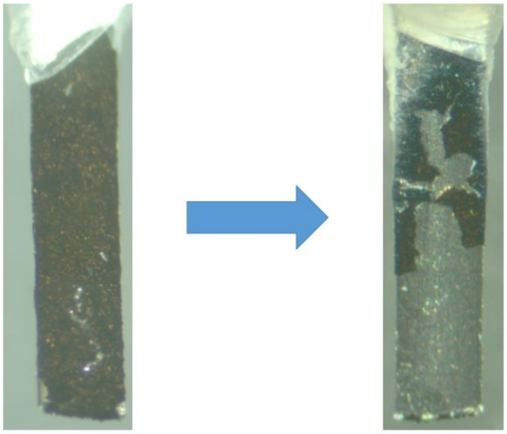
before
after

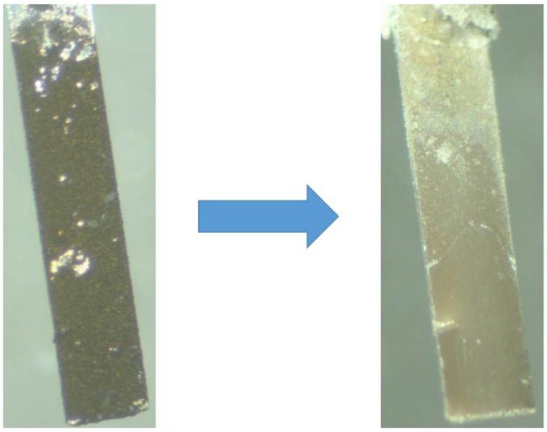
before
after

Figure S3. Optical microscope imagine before and after the electrochemical degradation. NIP, which was a) only stored in water for one month. NIP, which was degraded at an amplitude of b) 5 mV, d) 10 mV and f) 50 mV. MIP degraded at an amplitude of c) 5 mV, e) 10 mV and g) 50 mV.
